# Learning From Success or Failure? – Positivity Biases Revisited

**DOI:** 10.3389/fpsyg.2020.01627

**Published:** 2020-07-17

**Authors:** Tsutomu Harada

**Affiliations:** Graduate School of Business Administration, Kobe University, Kobe, Japan

**Keywords:** positivity biases, exploitation, exploration, asymmetric Q learning, asymmetric time-varying Q learning

## Abstract

The purpose of this study was to reexamine positivity learning biases through a Q learning computation model and relate them to behavioral characteristics of exploitation and exploration. It was found that while the positivity learning biases existed in the simple asymmetric Q learning model, they completely disappeared once the time-varying nature of learning rates was incorporated. In the time-varying model, learning rates depended on the magnitudes of success and failure. The corresponding positive and negative learning rates were related to high and low performance, respectively, indicating that successes and failures were accounted for by positive and negative learning rates. Moreover, these learning rates were related to both exploitation and exploration in somewhat balanced ways. In contrast, under the constant learning parameter model, positivity biases were associated only with exploration. Therefore, the results in the time-varying model are more intuitively appealing than the simple asymmetric model. However, the statistical tests indicated that participants eclectically selected between the asymmetric learning model and its time-varying version, a frequency of which differed across participants.

## Introduction

In economics, the direction of technological change has sometimes been viewed as biased, a phenomenon that is influenced by relative factors such as prices, history, or technical imbalances. All of these assume that an underlying learning process removes bottlenecks that hinder more profitable opportunities. The implicit assumption of these models is that learning is biased in specific directions – this claim has not been investigated rigorously in economics. In standard reinforcement learning (RL), the action values are assumed to be updated according to the reward prediction error (RPE), which is the difference between the actual reward and the expected reward; several studies have pointed out that the magnitude of a learning rate is biased depending on the sign of the RPE (Frank et al., 2007; Gershman, 2015, 2016). From a RL perspective, differential learning rates are represented by positive and negative RPEs.

This potential learning bias could be interpreted in terms of risk-seeking/aversion behaviors (Niv et al., 2012) or cognitive biases such as positivity bias and/or confirmation bias (Kuzmanovic and Rigoux, 2017; Lefebvre et al., 2017). Positivity bias describes the tendency to privilege positive news, while confirmation bias indicates the tendency to give more weight to outcomes that are consistent with one’s hypothesis (Palminteri et al., 2017). Numerous studies have indicated that differential learning rates tend to be biased in the direction of learning from positive RPEs, compared with negative RPEs (Frank et al., 2007; van den Bos et al., 2012; den Ouden et al., 2013; Aberg et al., 2015; Lefebvre et al., 2017). However, these studies did not fully explain how asymmetric learning rates are related to behavioral and cognitive properties.

Moreover, Katahira (2018) showed through simulation analysis that the autocorrelation of choice (i.e., the tendency to repeat the same choice or to switch to another choice irrespective of past outcomes) leads to pseudo-positivity biases and vice versa. Thus, without intrinsic autocorrelation, the RL model generates a statistical artifact leading to “pseudo-positivity bias” and “pseudo-confirmation bias.” Previous studies, therefore, have suggested that the positivity bias should be reexamined by removing the autocorrelation effects.

The purpose of this study was to investigate the determinants of learning biases in the asymmetric RL framework. In particular, we were interested in examining the relation between learning biases (Hills et al., 2015) and exploitation versus exploration. How does exploitation versus exploration affect learning biases? Although the related literature investigating learning biases does not consider these effects, we believe that the behavioral properties of exploitation and exploration play critical roles in human cognitive operations. This has been demonstrated by the success of RL in achieving higher performance than human beings in cognitive tasks (this does not imply the RL algorithms are superior to human cognitive systems). For example, in 2017, AlphaGo, a RL program that is applied to the board game Go, beat Ke Jie, the top-ranked player in the world at the time. AlphaGo consists of exploitation facets that suggest the best moves based on the knowledge obtained through a deep learning method. In addition, with certain probability, AlphaGo incorporates exploration aspects whereby the best moves that were suggested by the exploitation parts are designed to not be chosen; the purpose is to gain information with a view to figuring out new strategies to win the game. Thus, both exploitation and exploration facilitate learning in the AlphaGo program.

In creativity research, there is growing support for taking both divergent and convergent thinking into account (Gabora, 2010). Divergent thinking is defined as the ability to produce new approaches and original ideas by forming unexpected combinations from available information, and by applying abilities such as semantic flexibility, and fluency of association, ideation, and transformation (Guilford, 1967). Convergent thinking is defined as the ability to apply conventional and logical search, recognition, and decision-making strategies to stored information to produce an already known answer (Cropley, 2006). These two thinking processes could also be thought of in terms of exploitation and exploration. Exploitation refers to the optimization of current tasks under existing information and memory conditions, while exploration implies wider and sometimes random searches and trials that do not coincide with the optimal solutions provided by exploitation (see Sutton and Barto, 2018, for the trade-off between exploitation and exploration in the RL framework). Divergent thinking requires exploration rather than exploitation, whereby a wider search for a greater range of information should be undertaken. In contrast, convergent thinking seems to rely more on exploitation because the efficiency of search in a much narrower space should take full advantage of existing information.

In the Q learning model (see for example, Sutton and Barto, 2018), exploitation implies selection of the choices that yield the highest *Q* values, whereas exploration entails other non-optimal choices. Thus, both exploitation and exploration could be measured by the numbers of optimal and non-optimal choices, respectively. The present study assessed these measures to examine their effects on learning biases. We conjectured that exploration tends to be promoted more from good news and exploitation from bad news. On the one hand, exploration requires wider searches beyond current contexts, processes that could be bolstered by optimistic views generated by good news. That is, bad news seem more likely to discourage exploration. On the other hand, exploitation requires logical reasoning and deduction, so learning from bad news is essential for removing errors. Therefore, learning asymmetry exists between exploitation and exploration. Of course, it is also possible to argue that bad news could evoke exploration. Hence, using behavioral data, these hypotheses should be empirically tested.

First, we examined whether learning biases exist by estimating a standard Q learning model for the data obtained from the Iowa Gambling Task (IGT). Second, we related learning biases to performance in the IGT. The adaptive properties of asymmetric value updates have also been discussed in Cazé and van der Meer (2013); they showed that even in simple, static bandit tasks, agents with differential learning rates can outperform unbiased agents. Cazé and van der Meer (2013) suggested the existence of a situation in which the steady-state behavior of asymmetric RL models yields better separation of the action values compared with symmetric RL models. While this proposition was proved mathematically as asymptotic properties, real performance in cognitive tasks includes not only asymptotic properties but also transient outcomes (Katahira, 2018). Therefore, we tested empirically the relationship between learning biases and their performance in a cognitive task. Third, given these results, we examined the determinants of learning biases in terms of exploitation and exploration while controlling for related variables such as psychological personalities and working memory capacities.

Finally, a number of learning models have incorporated the modulation of learning rates (Daw et al., 2006; Behrens et al., 2007; Mathys et al., 2011); therefore, it is of critical importance to allow for this modulation. Thus, we incorporated the time-varying nature of learning rates in an asymmetric learning framework, and examined the learning biases.

## Materials and Methods

### Participants

A sample of 113 healthy undergraduate students at Kobe University (49 females, age range = 18–20 years, SD = 0.66) participated in the study. All participants were native Japanese with normal or correct-to-normal vision. The local Ethics Committee approved this study, and all participants signed an informed consent form before the experiment and were paid JPY 3,000 (approximately USD 28).

### Asymmetric Q Learning Model

**FIGURE 1:**
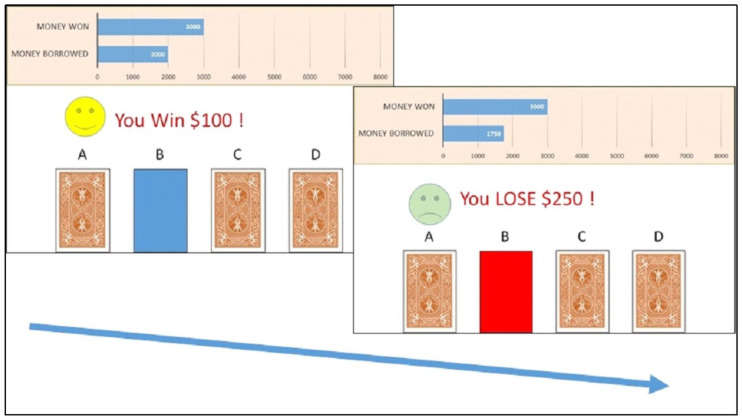
Example of trial in the Iowa Gambling Task in which the participant chose a card from Deck B. The participant received a reward of $100 **(left)**, immediately followed by a punishment of $250 **(right)**.

We adopted a Q learning framework (Sutton and Barto, 2018) to account for decision making in the IGT (Bechara et al., 1994). In the IGT, participants make a series of 100 choices from four decks of cards (see Figure 1). Two of the decks are advantageous and two of them are disadvantageous. The two disadvantageous decks always give rise to relatively high gains ($100) but also, with a 50% chance, to occasional large losses ($250), which results in an average loss of -$50 per trial. The two advantageous decks always generate lower gains each time ($50) but produce smaller losses ($50) with a 50% chance, resulting in an average gain of +$25 per trial. The goal is to maximize net scores across trials.

At each trial t, the action value *Q*_*i*_(*t*) of the chosen option (deck) i is updated via the following rule:

$${\text{Q}}_{i}\,\left(t+1\right)=\left\{ \begin{array}{c} {\text{Q}}_{i}\,\left(t\right)+\alpha^{+}\,\delta \,\left(t\right)+\phi \,i\,f\,\delta \,\left(t\right)\geq 0,\\ Q_{i}\,\left(t\right)+\alpha^{-}\,\delta \,\left(t\right)+\phi \,i\,f\,\delta \,\left(t\right)<0, \end{array}\right.\;\left(1\right)$$

with

$$\begin{array}{c} \delta \,\left(t\right)=R_{i}\,\left(t\right)-{\text{Q}}_{i}\,\left(t\right),\left(2\right) \end{array}$$

where R_*i*_(*t*)is the reward of the option i at trial t, and α^+^ and α^−^indicate the learning rates. ϕ is added in (1) as the choice trace to accounts for autocorrelation of choice which could affect the learning biases (Katahira, 2018). The updating equation (1) differs from the standard Q learning model in that the learning rates are assumed to be asymmetric between positive and negative RPEs. Our primary interest lies in the sign condition of α^+^−α^−^. If this is positive, it indicates that participants have learned more from success or good news, whereas the negative sign indicates they have learned more from failure or bad news. The RPE δ(*t*) is computed by subtracting the current value estimate from the obtained reward R. Participants thus update the action value estimate by scaling the prediction error with the learning rate and then adding this to the estimated value at the previous trial. Learning rates close to 1 indicate that a person has made fast adaptations based on prediction errors, and learning rates closer to 0 indicate slow adaptation. In the default setting, the initial action values are set to zero so that *Q*_*i*_(1) = 0 for i = 1,…,4.

For the unchosen option j (i≠j), the action value is updated as:

(1) $$Q_{j}\,\left(t+1\right)=Q_{j}\,\left(t\right)$$

Assume the chosen action at trial t is denoted by *a*(*t*) ∈ {1, 2, 3, 4}. The action value estimates of these four options are used to determine the probability of choosing either option. This probability is computed via the following softmax decision rule:

(2) $$P\left(a\left(t\right)=i\right)=\frac{e\,x\,p\,\left(\beta \,Q_{i}\,\left(t\right)\right)}{\sum_{j=1}^{4}e\,x\,p\,\left(\beta \,Q_{j}\,\left(t\right)\right)},$$

where *P*(*a*(*t*) = *i*) is the probability of choosing the action *a*(*t*) = *i* at trial t. The parameter β is the inverse temperature, a parameter that indicates the sensitivity of a participant’s choice to the difference in action value estimates.

The parameters of α and β in this model were estimated by optimizing the maximum *a posteriori* (MAP) objective function, that is, finding the posterior mode:

(3) $$\hat{\theta }=a\,r\,g\,m\,a\,x\,p\,\left(D_{s}|\theta_{s}\right)\,\text{p}\,\left(\theta_{s}\right),$$

where*p*(*D*_*s*_|θ_*s*_)is the likelihood of data *D*_*s*_ for subject *s* conditional on parameters θ_*s*_ = {α ^+ *S*^,α^−*S*^,β^*S*^}, and *p*(θ_*s*_)is the prior probability of θ_*s*_. We assume each parameter is bounded and use constrained optimization to find the MAP estimates. More specifically, since α is bounded between 0 and 1 and β takes non-negative values, their priors were assumed to follow beta distributions for α, and gamma distributions for β.

### Asymmetric Time-Varying Q Model

The asymmetric Q learning model is flexible in allowing for different learning rates from success and failure. However, it assumes these learning parameters are constant during the IGT for each participant. Following the time-varying Q learning model proposed by Pearce and Hall (1980) and Bai et al. (2014), we incorporated the time-varying learning parameters into the above asymmetric model in which *Q* values are updated according to

(4) $$\begin{array}{c} {\text{Q}}_{i}\,\left(t+1\right)=\left\{ \begin{array}{c} {\text{Q}}_{i}\,\left(t\right)+\alpha_{t}^{+}\,\delta \,\left(t\right)+\phi \,i\,f\,\delta \,\left(t\right)\geq 0,\\ Q_{i}\,\left(t\right)+\alpha_{t}^{-}\,\delta \,\left(t\right)+\phi \,i\,f\,\delta \,\left(t\right)<0, \end{array}\right. \end{array}$$

(5) $$\begin{array}{c} \alpha_{t+1}^{\pm }=\eta^{\pm }\,\left|\delta \,\left(t\right)\right|+\left(1-\eta^{\pm }\right)\,\alpha_{t}^{\pm } \end{array}$$

Now, the learning rate $\alpha_{t}^{\pm }$ depends on the absolute value of previous RPEs |δ(*t*)| and the constant parameter η controls the level of its influences. This asymmetric time-varying Q learning model differs from the previous model only in this updating characteristic, so equations (6)–(5) remain the same as before.

### Measures

This paper tested whether learning biases exist or not, and then, examined the determinants of learning biases, each learning parameter, and performance (total scores) in the IGT, which were used as dependent variables in the regression analysis. As explanatory variables, we used exploitation, exploration, its ratio (exploitation/exploration), the sum of RPEs, because our primary interest lies in the effects of exploitation and exploration on these dependent variables. As control variables that might affect learning biases, we used working memory capacities and personality scales.

#### Exploitation and Exploration

As measures for exploitation and exploration, we used the number of choices that exhibited the highest *Q* values and the number of choices that exhibited the lowest *Q* values, respectively. As related variables, the sum of RPEs δ(*t*) and the variance of the time-varying learning rate α_*t*_ were used, respectively, to measure the success and time flexibility of the underlying learning model. We also used the ratio of optimal and non-optimal choices to measure the relative strength of exploitation.

#### Working Memory Capacity (WMC)

Working memory capacity was measured using reading span, operation span, and matrix span tests, which are representative working memory tests (Conway et al., 2005). Reading span and operation span tests evaluate the capacity of verbal WMC and logical WMC, respectively, which in turn correspond to the phonological loop, according to Baddeley (2000). The matrix span test measures spatial WMC, corresponding to the visuo-spatial sketchpad in Baddeley’s model.

#### Big Five Scale of Personality

Big Five Scales (BFS) of personality traits are widely used to describe personality differences, which consist of five factors, namely openness to experience (inventive/curious vs. consistent/cautious), conscientiousness (efficient/organized vs. easy-going/careless), extraversion (outgoing/energetic vs. solitary/reserved), agreeableness (friendly/compassionate vs. challenging/detached), and neuroticism (sensitive/nervous vs. secure/confident) (Barrick and Mount, 1991; Miller, 1991; Piedmont et al., 1991). These scales were measured by 60 questions in Japanese, developed by Wada (1996). The scores were measured in descending order so that high scores in openness to experience, for example, imply lower openness to experience.

### Procedure

Participants completed reading span, operation span, matrix span tests (Conway et al., 2005), IGT, and BFS tests, which took approximately 60 min. This session was arranged for groups with a maximum of 50 participants in the presence of the instructor. The tests were performed on a 17′′ CRT monitor with PsytoolKit (Stoet, 2010, 2017). A break of at least 1 min was taken between the three tests. The order of these tests was randomly assigned in this session. In the following discussion, reading span, operation span, and matrix span test scores are denoted by verbal WMC, logical WMC, and spatial WMC, respectively.

### Learning Convergence

In the IGT, some of the participants eventually learned to keep picking the best (low risk, low return) decks. When participants remained in the best decks at least four times until the end of the game, we defined learning convergence took place for those participants. Based on this definition, we identified 60 participants who succeeded in learning convergence where the average number of trials before learning convergence was 70.9 (SD = 29.5).

The descriptive statistics of variables used in this study are reported in Table 1.

**TABLE 1:** Descriptive statistics.

	**Mean**	**SD**	**1**	**2**	**3**	**4**	**5**	**6**	**7**	**8**	**9**	**10**	**11**	**12**	**13**	**14**	**15**	**16**	**17**	**18**	**19**
1. α^+^−α^−^	0.15	0.32																			
2. α^+^	0.64	0.17	0.33**	**−−**																	
3. α^−^	0.64	0.17	0.32**	0.97**	**−−**																
4. Performance	2399.12	940.65	−0.46**	–0.12	−0.16^+^	**−−**															
5. Exploitation (Model 1)	43.73	16.39	–0.13	–0.08	–0.08	0.56**	**−−**														
6. Exploration (Model 1)	17.7	8.64	0.21*	0.06	0.06	−0.51**	−0.74**	**−−**													
7. Ratio (Model 1)	5.13	8.08	–0.11	−0.21*	−0.21*	0.47**	0.73**	−0.68**	**−−**												
8. RPE (Model 1)	–51.58	406.42	−0.17*	0.08	0.06	0.03	0.36**	−0.23**	0.07	**−−**											
9. Exploitation (Model 2)	39.84	13.83	0.10	0.14	0.10	0.33**	0.73**	−0.54**	0.72**	–0.10	**−−**										
10. Exploration (Model 2)	19.26	7.52	–0.08	–0.06	0.15^+^	−0.28**	−0.62**	0.50**	−0.60**	−0.56**	−0.73**	**−−**									
11. Ratio (Model 2)	3.19	3.97	–0.03	–0.09	–0.07	0.37**	0.61**	−0.48**	0.89**	0.80**	0.74**	−0.73**	**−−**								
12. RPE (Model 2)	13.95	117.44	0.14	0.15^+^	–0.09	−0.16^+^	–0.07	0.02	−0.24**	–0.07	0.04	0.04	–0.01	**−−**							
13. Extraversion	3.61	0.95	0.06	0.02	0.05	–0.03	–0.05	0.04	0.03	–0.05	0.03	0.10	0.04	0.10	**−−**						
14. Neuroticism	3.29	0.97	–0.10	–0.01	–0.06	0.09	–0.04	–0.06	0.05	0.11	–0.08	0.00	0.00	–0.04	−0.40**	**−−**					
15. Openness	3.89	0.83	0.01	0.02	0.06	–0.01	–0.05	0.08	0.04	–0.09	–0.04	0.08	0.01	0.09	0.23*	−0.21*	**−−**				
16. Conscientiousness	4.19	0.69	–0.14	−0.19*	−0.22**	0.17*	0.12	−0.16^+^	0.13	0.10	–0.04	–0.09	0.11	–0.13	0.08	0.10	0.04	**−−**			
17. Agreeableness	3.4	0.89	–0.01	–0.01	0.03	−0.24**	−0.18*	0.23**	–0.05	−0.30**	–0.10	0.17*	–0.04	0.00	0.37**	−0.24**	0.14	0.29**	**−−**		
18. Spatial WMC	23.81	13.38	–0.15	0.08	0.04	0.17*	0.14	–0.07	0.05	0.12	0.08	0.14	–0.07	0.03	0.04	0.00	–0.10	0.01	0.02	**−−**	
19. Verbal WMC	25.73	12.75	–0.10	0.09	0.06	0.16^+^	0.07	–0.10	–0.01	0.07	0.01	0.16^+^	–0.14	–0.03	0.05	–0.07	–0.02	0.01	–0.08	0.37**	**−−**
20. Logical WMC	28.18	11.65	0.16^+^	0.03	0.02	0.06	0.11	–0.07	0.08	0.12	0.05	0.06	–0.06	0.02	–0.02	0.01	0.07	–0.01	–0.02	0.22*	0.24**

+*p* < 0.10, *p < 0.05, **p < 0.01. RPE refers to the sum of reward prediction errors. Model 1 and Model 2 indicates, respectively, an asymmetric Q learning model and an asymmetric time-varying Q learning model.

## Results

### Positivity or Negativity Biases

**FIGURE 2:**
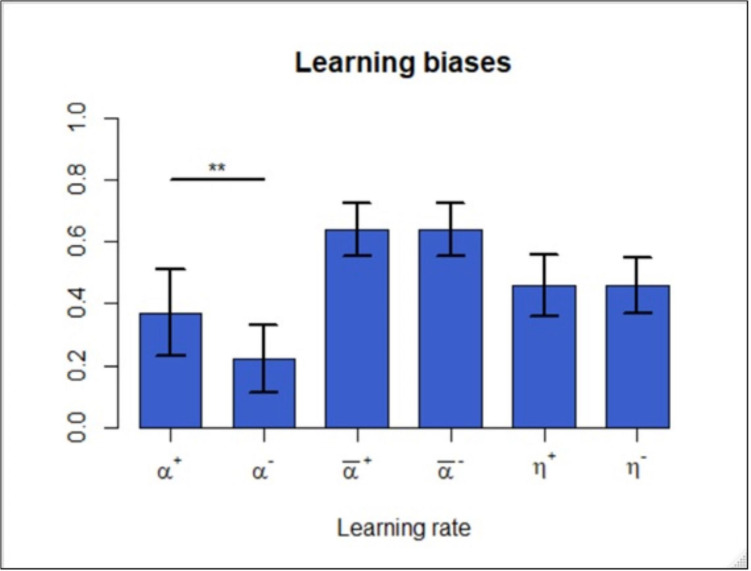
Learning rate analysis of the asymmetric Q learning model (left) indicated that learning was positively biased. Learning rate analysis of the asymmetric time-varying Q learning model (middle and right) indicated that the average learning rates (${\bar{\alpha }}^{+}$ and ${\bar{\alpha }}^{-}$) and the underlying learning parameters (η^+^ and η^−^) did not yield significant differences. **p < 0.01.

To examine the existence of positivity and negativity biases, we estimated learning rates in the asymmetric Q learning and its time-varying version. In the former model, we found that the positive learning rate was significantly higher than the negative one [*T*(224) = 4.49; *P* = 1.13e-05] (Figure 2, left), which is consistent with related studies. However, in the asymmetric time-varying version, the biases disappeared in terms of the differences between the average values of ${\bar{\alpha }}^{+}$ and ${\bar{\alpha }}^{-}$ [*T*(224) = −0.07; *P* = 0.94] (Figure 2, middle). The averages were taken here because these variables change over time for each participant as specified in (7). In addition, we also examined the difference between η^+^ and η^−^, yet no statistical significance was observed between the two [*T*(224) = −0.19; *P* = 0.85] (Figure 2, middle). In contrast to the former model, the signs of the differences in this model became negative in ${\bar{\alpha }}^{\pm }$ and η^±^, though they were not significant. Note that in the subsequent analysis, since η^±^ did not exert statistically significant effects, we henceforth considered only ${\bar{\alpha }}^{\pm }$ in the time-varying asymmetric model.

Thus, in the standard asymmetric model, even after controlling for pseudo bias arising from the autocorrelation, we still confirmed the positivity biases. Once we allowed for the time-varying nature of learning rates, however, the biases, whether they were positive or negative, no longer emerged.

### Effects on Performance

Given the positivity biases in the asymmetric Q learning model as indicating a general tendency to respond more to positive RPEs, a question arises as to how this bias relates to performance in the IGT. To examine the effects of the positivity bias on performance, we regressed α^+^, α^−^ and its difference on the total sum of rewards in the IGT. The results are presented in Table 2.

**TABLE 2:** Effects of learning parameters on performance (SE in parentheses).

**Variables**	**(1)**	**(2)**
**Constant Terms**	2602.3** (87.5)	2543.7** (144.9)
α^+^−α^−^	−1341.3** (247.2)	
α^+^		−1266.1** (289.0)
α^−^		1478.2** (366.5)
**R-squared**	0.21	0.212

**p < 0.01.

It may be surprising that both α^+^ and α^+^−α^−^ had negative effects, whereas α^−^ had positive effects, on performance. This implies that the positivity bias did not contribute to higher performance. Instead, the negativity bias was required to achieve higher rewards. This result was consistent with the stochastic structure of the IGT setting because the expected rewards were higher from low risk, low return decks than from high risk, high return decks. Hence, participants should be more sensitive to negative RPEs and quickly converge to low risk, low return decks. Despite this stochastic structure of the IGT, participants who were not informed of these stochastic properties showed a significant tendency toward preferential learning at the risk of losing rewards.

In the asymmetric time-varying model, since the learning biases did not exist, we examined the effects of each learning parameter on IGT performance. The results are shown in Table 3. In contrast to the previous results, ${\bar{\alpha }}^{+}$ was positively related to performance, while ${\bar{\alpha }}^{-}$ exerted negative effects on performance. Hence, these effects were completely opposite to the previous results on α^+^ and α^−^. Note that the magnitude of ${\bar{\alpha }}^{-}$ depends on that of failure as specified in (7). This implies that the greater loss in rewards was reflected in the learning parameter ${\bar{\alpha }}^{-}$, whereas the greater gain was absorbed in ${\bar{\alpha }}^{+}$. Consequently, ${\bar{\alpha }}^{+}$ and ${\bar{\alpha }}^{-}$ are related to performance, as shown in Table 3.

**TABLE 3:** Effects of learning rates on performance (SE in parentheses).

**Variables**	
**Constant Terms**	2917.8** (347.6)
α^+^	4440.9^+^ (2321.1)
α^−^	−5237.8* (2326.4)
**R-squared**	0.06

+*p* < 0.10, *p < 0.05, **p < 0.01.

### Determinants of Learning Rates

The results showed that the positivity bias disappeared once the time-varying nature of learning parameters was incorporated. This stands in sharp contrast to related studies in which positivity biases were exhibited. To further examine the differences between positivity biases and no biases in the two models, we evaluated the effects of exploitation and exploration on these learning parameters while controlling for personality and working memory characteristics of the participants.

First, we examined the effects of exploitation and exploration on the positivity biases, α^+^−α^−^, in the asymmetric Q learning model. The results are shown in Table 4.

**TABLE 4:** Determinants of positivity biases (SE in parentheses).

**Variables**	
**Constant Terms**	0409** (0.373)
**Exploitation**	−0001** (0.003)
**Exploration**	0.012^+^ (0.007)
**Exploitation/Exploration**	0.002 (0.003)
**Reward Prediction Errors**	0.000
**Exraversion**	0.029 (0.036)
**Neuroticism**	−0.038 (0.035)
**Openness**	−0.021* (0.037)
**Conscientiousness**	−0.04 (0.046)
**Agreeableness**	−0.05** (0.041)
**Spatial WMC**	−0.003 (0.002)
**Verbal WMC**	−0.003 (0.003)
**Logical WMC**	0.006 (0.003)
**R-squared**	0.18

+*p* < 0.10, *p < 0.05, **p < 0.01.

The results showed that positivity bias was not directly related to personalities. Instead, it was accounted for by exploration, the sum of RPEs, and logical WMC. Note that exploration indicates the selection of the decks with the lowest *Q* values. The question arises as to how they selected these least optimal choices in terms of the underlying learning model. The result suggests that this was facilitated by the positivity bias. That is, participants responded more to the choices that generated high rewards, and tended to repeat the same choices. However, since the IGT was designed to set high risk, high return decks with lower expected rewards, these choice patterns led to the selection of the decks with the lowest *Q* values. Hence, exploration was related to the positivity bias, although they did not give rise to higher performance. This interpretation was supported by the negative effect of RPEs, indicating that participants showing the positivity bias were more likely to lower their sum of RPEs.

Logical WMC was positively related to the positivity bias. This logical WMC was measured by the capacity to memorize the results of the previous mental calculation while undertaking current calculation tasks. In the IGT, participants with a positivity bias have to remember the results of the past four decks, in particular, those generating higher rewards. Logical WMC facilitated these cognitive operations, leading to its positive effects on the positivity bias.

Next, we examined the effects of exploitation and exploration on the learning parameters, ${\bar{\alpha }}^{\pm }$, in the asymmetric time-varying model. In this case, since no significant learning biases had been identified, we examined the determinants of each learning parameter. Because the learning parameters only take values between 0 and 1, we transformed these into

(6) $$\begin{array}{c} \tilde{\alpha }=l\,o\,g\,\frac{\alpha }{1-\alpha }, \end{array}$$

where the original regression model is assumed to be α = 1/(1−exp(−*X*)) and *X* indicates explanatory variables. With this log transformation, the ordinary least squares (OLS) generates statistically consistent estimates. The results are shown in Table 5.

**TABLE 5:** Determinants of learning parameters (SE in parentheses).

**Variables**	**α^+^**	**α^−^**
**Constant Terms**	1.124 (1.220)	1.339 (1.171)
**Exploitation**	0.021^+^ (0.012)	0.020^+^ (0.012)
**Exploration**	−0.036 (0.023)	−0.042^+^ (0.022)
**Exploitation/Exploration**	−0.112* (0.041)	−0.115** (0.040)
**Reward Prediction Errors**	0.001 (0.001)	0.001 (0.001)
**Exraversion**	0.035 (0.121)	0.052 (0.116)
**Neuroticism**	0.056 (0.115)	0.034 (0.110)
**Openness**	0.050 (0.124)	0.087 (0.119)
**Conscientiousness**	−0.315^+^ (0.156)	−0.398** (0.150)
**Agreeableness**	0.116 (0.128)	0.200 (0.123)
**Spatial WMC**	0.006 (0.008)	0.003 (0.008)
**Verbal WMC**	0.006 (0.009)	0.004 (0.008)
**Logical WMC**	−0.001 (0.009)	−0.002 (0.008)
**R-squared**	0.16	0.21

+*p* < 0.10, *p < 0.05, **p < 0.01.

As expected from the results of no learning biases, both ${\bar{\alpha }}^{+}$ and ${\bar{\alpha }}^{-}$ showed quite similar patterns. Exploitation had positive effects whereas the ratio of exploitation and exploitation exhibited negative effects. This implies that exploitation was positively related to ${\bar{\alpha }}^{+}$ and ${\bar{\alpha }}^{-}$, yet exploration exerted positive effects, relative to exploitation. However, in ${\bar{\alpha }}^{-}$, exploration itself had negative effects, although its significance level was lower than that of the ratio of exploitation and exploitation. Therefore, in ${\bar{\alpha }}^{+}$ and ${\bar{\alpha }}^{-}$, the net effects of exploitation and exploration were positive.

These results suggest that on the one hand, the positivity biases were positively related to exploration. On the other hand, however, the learning parameters in the time-varying model were balanced in terms of reflecting exploitation and exploration. Therefore, incorporating the time-varying nature of learning parameters that reflect the magnitude of success or failure led to counting on both exploitation and exploration, rather than depending exclusively on either exploitation or exploration. Under these conditions, the positivity or negativity biases disappeared so that learning rates from success and failure exhibited similar magnitudes.

### Model Fits

To compare the above two models, we calculated the Bayes factors on an individual basis following the criteria suggested by Kass and Raftery (1995). According to this criteria, 55 participants were selected in favor of the simple asymmetric model and 54 participants in favor of the time-varying asymmetric model. This implies that the models cannot be differentiated statistically. Therefore, we cannot completely deny the positivity biases in this study. What is implied in this analysis is the role of time-varying assumption on learning parameters in generating results of no positivity biases.

## Discussion

In this study, we found that participants displayed a positivity bias in the IGT, even though it was related to lower performance, as far as the simple asymmetric Q learning model was concerned. However, once the time-varying nature of learning rates was added to the model, learning biases – whether positivity or negativity – were completely eliminated. In contrast to related studies, in particular those exhibiting positivity biases (Frank et al., 2007; van den Bos et al., 2012; den Ouden et al., 2013; Aberg et al., 2015; Lefebvre et al., 2017; Palminteri et al., 2017), we could not identify such learning biases in our study. Although the pseudo positivity bias could emerge with the autocorrelation of choices (Katahira, 2018), this study still found the existence of the positivity bias after controlling for the autocorrelation effects. Only after controlling for the time-varying nature of learning rates did the biases disappear, implying that the pseudo positivity bias could also emerge from the time-dependency of learning rates.

Palminteri et al. (2017) showed through experiment that the positivity bias could be interpreted as the confirmation bias, which implies that participants preferentially took into account the outcomes that confirmed their current behavioral policy and discounted the outcomes that contradicted it. Furthermore, they suggested that these learning biases can be maladaptive in the context of learning performance, but can serve as adaptive in other cognitive domains, thus generating a net adaptive value. Indeed, some studies have demonstrated the relation between optimism and high adaptive values (MacLeod and Conway, 2005; Tindle et al., 2009; Carver et al., 2010; Johnson and Fowler, 2011). Regarding the more specific context of optimism in RL, Cazé and van der Meer (2013) showed that in low-reward environments, an agent learns asymmetrically in an optimistic manner. As a result, they speculated that positivity or confirmation biases promote self-esteem and confidence, and have overall favorable real life outcomes (Weinstein, 1980).

However, these results presupposed, as in our asymmetric Q learning model, that learning parameters remained constant during the experiments. It is more likely that participants change how much to learn from success or failure, depending on the magnitudes of each. If participants face huge successes (failures), they will significantly improve (decrease) the *Q* values of the corresponding decks. However, if the gains (loss) are modest, the improvement (reduction) remains modest.

The positivity biases observed in the asymmetric Q learning model in our study seemed to reflect the underlying stochastic structure of the game, rather than an adaptive strategy that promotes self-esteem and confidence. In our study, for instance, higher rewards were expected when participants kept selecting low risk, low return, instead of high risk, high return, decks. Although learning rates might differ across participants, they could have converged to the former choices sooner or later during the game. Once they reached the steady state of choosing the same decks, more frequent gains were expected, and they did not switch to different decks, implying that high *Q* values were put on the corresponding decks. Hence, the biases might have been caused by the convergence to and discovery of low risk, low return decks. This might also account for why the positivity and confirmation biases suggested in Palminteri et al. (2017) induced overall favorable real life outcomes. The causality was not positivity biases toward high performance; rather, high adaption to remaining in steady states led to the generation of positivity biases because steady states imply repeating the same choices over time, which induces higher *Q* values with more frequent success.

However, before reaching a steady state, a number of failures take place. Indeed, the results for the determinants of performance in our study indicated that the positivity biases in the asymmetric learning model were related to lower performance. This suggests that the positivity biases, under the constancy of learning parameters, reflected a series of failures before participants reached their steady states, leading to negative effects on performance. Once the time-varying nature of learning rates was added, the positive (${\bar{\alpha }}^{+}$ and η^+^) and negative learning parameters (${\bar{\alpha }}^{-}$ and η^−^), respectively, accounted for high and low performance. As stated above, the magnitude of ${\bar{\alpha }}^{-}$ depends on that of failure as specified in (7) so that more loss was reflected in the learning parameter ${\bar{\alpha }}^{-}$ whereas more gain was absorbed in ${\bar{\alpha }}^{+}$. Similar reasoning could also be applied to η^+^ and η^−^. Therefore, under more flexible, time-varying learning parameters, the positive and negative learning parameters, respectively, follow success and failure without biases. The positivity biases were caused by converging to steady states, yet exhibited negative effects on performance because of the search phase before reaching steady states in which a number of failures was expected.

We also examined the relationship between positivity biases and dynamic policies of exploitation and exploration. The results indicated that the positivity biases were more related to exploration, suggesting that they were associated with information-gathering activities at the sacrifice of optimization. If the confirmation biases are correct, it follows that some confidence is required to select seemingly unfavorable choices with the aim to collect information. Obviously, exploitation alone easily gets stuck with local optimums. To escape from this sub-optimal state, wide information searches beyond current contexts are necessitated, which corresponds to exploration. However, in the time-varying learning parameters, both positive and negative learning rates were related to exploitation and exploration simultaneously. This indicates that participants showed a balanced cognitive tendency toward exploitation and exploration, rather than exclusively toward exploration at some sacrifice of exploitation. This balance seems to generate no learning biases under the time-varying learning parameters. Obviously, some balance between exploitation and exploration is required to enhance the adaptive value even in broader cognitive contexts.

It has been proposed that organisms can change their behavioral patterns flexibly by choosing actions on the basis of on their expected returns (Dayan and Abbott, 2001; Bogacz, 2007; Sutton and Barto, 2018). However, the present study indicates that human beings not only determine their behavioral patterns according to the expected returns, but also consider information sampling as exploration. The balance between optimization and information acquisition is a key to higher adaptive values.

In this respect, it should be noted that compared with related studies that adopt two-armed bandit games, our study used the IGT in which four alternatives were presented to participants because an increase in the number of alternatives could have had non-negligible effects on exploration. The related studies show that an increase in the number of choice alternatives can reduce the probability that one of the alternatives will be selected (Iyengar and Lepper, 2000; Boatwright and Nunes, 2001). This is because adding choices increases choosers’ confusion (Huffman and Kahn, 1998; Iyengar and Lepper, 2000) and leads to weaker preferences (Dhar, 1997; Iyengar and Lepper, 2000; Chernev, 2003; Gourville and Soman, 2005), which in turn leads to increases in risk-seeking (Ert and Erev, 2007). This implies that the IGT arguably encourages more exploration than the two-armed bandit game. Indeed, in the simple asymmetric learning model, only exploration accounted for learning parameters. Nevertheless, once the time-varying nature of learning parameters was allowed, exploitation, as well as exploration, mattered in determining the magnitude of learning parameters. Therefore, participants seem to balance exploitation against exploration, even if increases in the number of choices induce the latter. It would be interesting to examine how the effects of exploitation and exploration are altered as the number of alternatives increases beyond four.

It appears that the asymmetric time-varying model is better than the simple asymmetric one because learning seems to reflect not only success and failure, but also their magnitudes. Regardless of success or failure, if the magnitudes are sufficiently large, they should significantly affect subsequent choice behaviors, indicating substantial updating of corresponding *Q* values in our framework. Hence, it would be more intuitive and reasonable to assume that humans learn more from huge, rather than modest, successes or failures. The standard asymmetric learning model failed to incorporate this learning feature. In the time-varying version, learning rates were associated with both exploitation and exploration in balanced manners, results that are also intuitively appealing.

However, when we calculated the Bayes factors and compared the two models on an individual basis following the criteria suggested by Kass and Raftery (1995), 55 participants selected the simple asymmetric model and 54 chose the time-varying asymmetric model. This implies that the models cannot be differentiated statistically. Therefore, it seems more reasonable to assume that participants eclectically selected either model, the frequency of which varied across participants. Thus, this study proposed the alternative model with no learning biases, as opposed to the standard asymmetric Q learning model with the positivity biases. In reality, the participants seemed to switch between the two models.

Finally, a remark is deserved for the result that personality characteristics had almost no effects on learning parameters, not only in the asymmetric learning model but also in the time-varying version. Behavioral, cognitive, and emotional characteristics are defined as personality (Corr and Matthews, 2009). However, valence-induced learning had nothing to do with personality characteristics in this study. This suggests that learning rates underlie the learning system in human brains at a more subconscious level. Thus, we expect that learning is built into the neural system to facilitate exploitation and exploration so as to improve the adaptive value in broader cognitive contexts. In particular, in the time-varying model, each underlying learning parameter showed close association with both exploitation and exploration. In the current study, the participants were not informed of the underlying stochastic structure in the IGT. In uncertain situations, the participants implemented their innate learning tendencies to put a balanced emphasis on success and good news, rather than either bias, inducing both exploration and exploitation in their cognitive operations. We conjecture that after uncertainty is reduced through learning, they might change this balance to put more weight on exploitation, reflecting a stable environment. This adaption to uncertainty through learning via exploitation and exploration was shared by participants, regardless of personality characteristics.

## Conclusion

By investigating learning biases through computation models, the current study demonstrated that while positivity learning biases existed in the simple asymmetric Q learning model, even after controlling for autocorrelation effects, they completely disappeared once the time-varying nature of learning rates was incorporated. In the time-varying model, learning rates depend on the magnitude of success and failure. If gains or losses are large, *Q* values are sufficiently updated to reflect such magnitudes. The corresponding positive and negative learning rates were related to high and low performance, respectively, indicating that successes and failures were accounted for by positive and negative learning rates. Moreover, it was found that these learning rates were related to both exploitation and exploration in somewhat balanced ways. Thus, positive and negative learning rates, respectively, in charge of success and failure, simultaneously take into account exploitation and exploration. In contrast, under the constant learning parameter model, positivity biases were associated only with exploration. Therefore, results in the time-varying model are more intuitively appealing.

However, the statistical tests indicated that we cannot differentiate between the two models statistically. Therefore, the positivity or confirmation biases found in the simple asymmetric model cannot completely be denied. Nevertheless, the current study at least highlighted that the results were sensitive to the assumption of the constancy of learning parameters. This does not imply that the question of asymmetric learning rates was resolved.

Obviously, our results critically depended on functional specifications such as in (4) and (7). To make the results comparable with those in the related study, we retained them in the current study. However, it could be one of our future challenges to consider alternative functional specifications and examine how the results are altered.

Thus, further studies are needed to figure out what determines the switch between the two models and the resulting positivity or no learning biases. Moreover, neural correlates of exploitation and exploration that are expected to have critical effects on learning biases should be examined. This also constitutes one of our future research challenges.

## Data Availability Statement

The datasets generated for this study are available on request to the corresponding author.

## Ethics Statement

The studies involving human participants were reviewed and approved by the Ethics Committee, Graduate School of Business Administration, Kobe University. The patients/participants provided their written informed consent to participate in this study.

## Author Contributions

The author confirms being the sole contributor of this work and has approved it for publication.

## Conflict of Interest

The authors declare that the research was conducted in the absence of any commercial or financial relationships that could be construed as a potential conflict of interest.
